# Different Influences of Rare Earth Eu Addition on Primary Si Refinement in Hypereutectic Al–Si Alloys with Varied Purity

**DOI:** 10.3390/ma12213505

**Published:** 2019-10-25

**Authors:** Feng Mao, Shizhong Wei, Liming Ou, Cheng Zhang, Chong Chen, Xiaodong Wang, Zhiqiang Cao

**Affiliations:** 1 National Joint Engineering Research Center for Abrasion Control and Molding of Metal Materials, Henan University of Science and Technology, Luoyang 471003, China; maofeng718@163.com (F.M.);; 2 School of Mechanical Engineering, Yangtze University, Hubei 434023, China; 3 Key Laboratory of Solidification Control and Digital Preparation Technology (Liaoning Province), School of Materials Science and Engineering, Dalian University of Technology, Dalian 116024, China

**Keywords:** primary Si, rare earth, modification, hypereutectic Al–Si alloy, mechanical properties

## Abstract

The effect of alloying the Eu element on primary Si refinement in varied purity Al–16Si alloys was studied by scanning electron microscopy (SEM), thermal analysis, micro x–ray diffraction (μ–XRD), electron probe microanalysis (EPMA), and transmission electron microscopy (TEM). The results indicate that the P impurity element in hypereutectic Al–Si alloys has a great influence on the rare earths’ refinement efficiency of primary Si. Coinstantaneous primary Si refinement and eutectic Si modification by Eu was obtained in high purity (HP) Al–16Si and commercial purity (CP) Al–16Si–0.06P alloys, but the primary Si was gradually coarsened in CP Al–16Si alloys. An excellent integration of ultimate tensile strength (144.8 MPa) and elongation (9.8%) of CP hypereutectic Al–16Si–0.06P alloy was obtained by adding 0.15% Eu. The refinement of primary Si in Eu–modified HP Al–16Si alloys was related to the constitutional undercooling of Eu. There was no sufficient Eu element partition into the primary Si particles, and fewer parallel twins, rather than multiple twins, were observed within them. The refinement of primary Si in CP Al–16Si–0.06P alloys was caused by the overlay of two kinds of mechanisms including the heterogeneous nucleation mechanism of AlP and the constitutional supercooling mechanism of Eu. However, in order to refine the primary Si in CP hypereutectic Al–16Si alloys, the Eu:P weight ratio should not exceed 3.33, otherwise the refinement efficiency of primary Si will be reduced due to mutual poisoning between Eu and P. This work can be used to interpret the controversy concerning the influence of rare earths on the primary Si in hypereutectic Al–Si alloys, thereby elucidating the importance of alloy purity to primary Si refinement by rare earths.

## 1. Introduction

Hypereutectic Al–Si alloys are widely used to produce pistons, engine blocks, cylinder heads, brake fraction plates, and rocker arms in the aerospace and automotive industry due to their low weight, low thermal expansion coefficient, excellent wear resistance, and castability [1,2]. It is common knowledge that the mechanical properties of Al–Si alloys are closely associated with the shape and size of the Si phase [3]. Nevertheless, the stress concentration is easily generated by the coarse irregularly shaped primary Si and plate–like eutectic Si, which can adversely affect the mechanical properties of Al–Si alloys, particularly the plasticity [4,5]. In order to achieve good mechanical properties, the primary Si refinement and eutectic Si modification must be achieved simultaneously, which can be realized by several methods including rapid solidification [6], melt overheating [7], ultrasonic vibration [8], electromagnetic field [9], and chemical modification [10]. In these technologies, chemical modification has found wide application in the industry due to the advantages of simple operation and low cost.

Effective refinement of primary Si in hypereutectic Al–Si alloys has been frequently obtained by adding AlP particles, which can act as the heterogeneous nuclei of primary Si crystals [11,12,13]. Recently, a solidification sequence map for a P–refined hypereutectic Al–Si alloy was framed by S.M. Liang et al. [14], which showed that AlP particles occurred before or during the precipitation of primary Si with a P content in excess of 8 ppm. Several kinds of Al–P master alloys [15,16,17] have been broadly used for hypereutectic Al–Si alloys due to their wonderful refining capability on primary Si. However, the P element had no effect on the size and morphology of eutectic Si [18]. With respect to eutectic Si, Na and Sr elements [19,20] have been successfully used in industry production, which has efficiently transformed eutectic Si from plate–like to a fibrous structure. Nevertheless, they have little influence on the dimension of primary Si in hypereutectic Al–Si alloys [21,22]. Unfortunately, after adding P and Na/Sr to the melt simultaneously, the refinement efficiency of primary Si will be reduced due to the mutual poisoning between them [23,24].

In recent years, it has been reported that several rare earth elements are able to achieve primary Si refinement and eutectic Si modification simultaneously in hypereutectic Al–Si alloys [3,25,26,27,28]. Moreover, rare earths have a high efficiency of degassing and slag–removal in aluminum alloys with the characteristic of being environmentally–friendly, thus making them a novel modifier with broad potential industrial application. However, there is still controversy concerning the influence of rare earths on the primary Si in hypereutectic Al–Si alloys. Q.L. Li et al. [25] found that the addition of 1.0% Ce significantly refined the primary Si and transferred the morphology from coarse irregular to fine blocky. However, J.C. Weiss et al. [29] and M. Shafei et al. [30] reported that rare earth Ce had no influence on the refinement of primary Si in hypereutectic Al–Si alloys. Q.L. Li et al. [3] showed that the addition of 0.8% Y element produced a 62.9% drop in the average size of primary Si and a 38.6% drop in the aspect ratio, while B.D. Sun et al. [31] reported that Y could not refine primary Si without other additives, but that the primary Si was further refined by the combined additions of P and Y. M.F. Kilicaslan et al. [32] observed that rare earth Sc refined the primary Si without dramatically changing the morphology in Al–20Si alloys. Nevertheless, P. Chokemorh et al. [33] found that the addition of Sc inhibited the precipitation of primary Si in Al–20Si alloys because of the mutual poisoning between Sc and AlP. Furthermore, P is a common impurity in commercial purity (CP) Al alloys and the interaction between P and rare earth has been frequently reported in hypoeutectic Al–Si alloys by forming binary phosphides (such as YbP [34], YP [35], and ScP [36]). Thus, the alloy purity should be the key factor to affect the primary Si refinement by rare earths. However, the effect of alloy purity on the rare earth refinement efficiency of primary Si in hypereutectic Al–Si alloys has not been reported to date.

K. Nogita et al. [37] studied the fourteen rare earth elements’ modification efficiency of eutectic Si in the Al–10Si alloy. It was discovered that only Eu could produce fibrous eutectic Si, showing the strongest modification efficiency of eutectic Si among the rare earth elements, similar to Sr and Na [38]. The addition of 0.1% Eu was also reported to enhance the tensile properties of A356 alloys in our previous work [39]. However, the influence of Eu on the primary Si in hypereutectic Al–Si alloys is still unclear. Therefore, the purpose of this work was to study the influences of Eu addition on primary Si refinement in high purity (HP), commercial purity (CP), and P–refined commercial purity (CP) hypereutectic Al–16Si alloys and clarify the refinement mechanisms in varied purity alloys according to the experimental results. Furthermore, the tensile properties of Eu–modified CP hypereutectic Al–16Si alloys were also measured to evaluate the effectiveness of modification. This work will not only elucidate the importance of alloy purity to primary Si refinement by rare earths, but will also develop a new modifier of hypereutectic Al–Si alloys for engineering applications.

## 2. Experimental Details

Three kinds of high purity (HP) Al–16Si, commercial purity (CP) Al–16Si, and CP Al–16Si–0.06P alloys were used as the base alloys. The HP Al–16Si base alloy was produced by melting HP Al (99.99%) and HP Si (99.996%), while the CP Al–16Si and CP Al–16Si–0.06P base alloys were prepared with the CP Al (99.7%), CP Si (99.3%), and Al–5P master alloy. Table 1 shows the chemical compositions of the three base alloys analyzed by glow discharge mass spectrometer (GDMS, Finnigan ELEMENT GD, MA, USA). Next, the base alloys were melted in a resistance furnace at 850 °C. An Al–6Eu master alloy was added into some melts with 30 min holding. After degassing and slag–removal, the melt of 750 °C was cast into a stainless steel mold (Φ30 mm × 70 mm). The nominal added Eu contents for each alloy are also listed in Table 1.

A K type thermocouple was placed in the center of a graphite mold with the tip 25 mm from the bottom, and the cooling curves from the thermocouple were measured using a temperature recorder with a sampling step of 200 milliseconds during solidification [39]. The samples were ground using SiC paper up to 1500 mesh and then polished with 1 um diamond paste. The samples after polishing were etched with 0.5% HF and 15% HCl solutions to examine the two–dimensional (2D) and three–dimensional morphologies (3D) of primary Si, respectively. The average size of primary Si was calculated with Image–Pro Plus 6.0 software (Media Cybernetics, Rockville, MD, USA). Due to the same grey–level between primary Si and eutectic Si, all the primary Si particles were manually distinguished. On each sample, twenty fields with a magnification of 1500 times were examined, then the average particle size of primary Si was calculated as follows:

(1) $$\mathrm{Particle}\;\mathrm{size}=\;\frac{1}{m}\overset{m}{\underset{j=1}{\sum }}{\left(\frac{1}{n}\overset{n}{\underset{i=1}{\sum }}D_{i}\right)}_{j}$$

where *Di* is the average length of diameters measured at 2 degree intervals and passing through the particle’s centroid; *n* is the number of particles of a single field; and m is the number of the fields. The micro x–ray diffraction (μ–XRD) was performed at beamline BL15U1 at the Shanghai Synchrotron Radiation Facility (SSRF) (Shanghai, China) and the correlative experimental details were described in our preceding paper [40]. The microstructures were characterized by scanning electron microscopy (SEM, Zeiss supra 55, Oberkochen, Germany) operated at 15 KV, electron probe microanalysis (EPMA–1600, Shimadzu, Kyoto, Japan) operated at 15 KV, and transmission electron microscopy (TEM, aberration–corrected FEI Titan G2 60–300, Hillsboro, OR, USA) operated at 200 kV. The tensile specimens were prepared as per the ASTME8M–04 standard with a gauge length of 30 mm and a gauge diameter of 6 mm. The tensile test was conducted with a strain rate of 1.25 × 10^−3^ s^−1^ at room temperature using a universal tensile testing machine (Instron 5500R, Canton, UK). The values of ultimate tensile strength (UTS) and elongation (EI) were calculated by the average of three tests for each alloy and the fracture surfaces of the alloys were also characterized by SEM.

## 3. Results and Discussion

### 3.1. Microstructure Evaluation

**Figure 1:**
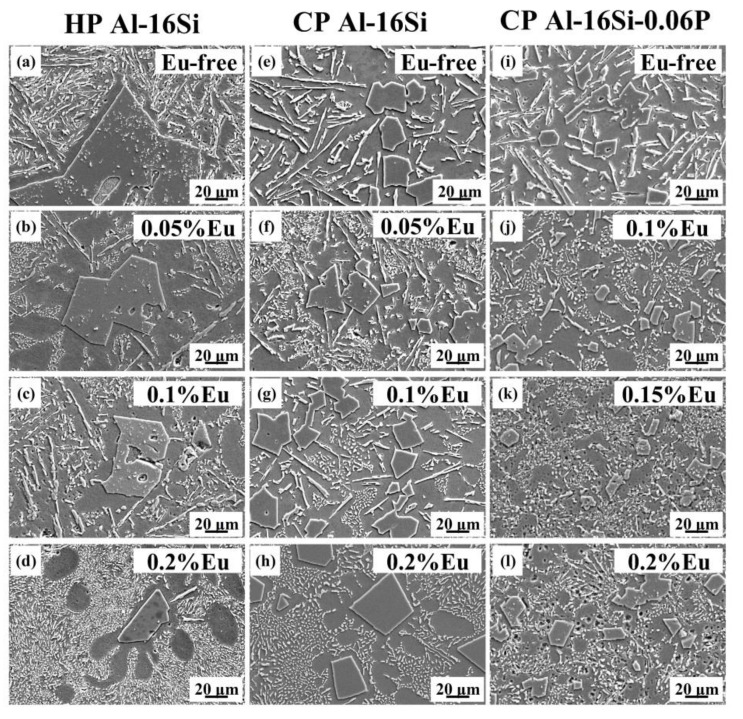
The microstructures of three series of HP Al–16Si, CP Al–16Si ,and CP Al–16Si–0.06P alloys with different Eu concentrations: (**a**) alloy A; (**b**) alloy B; (**c**) alloy C; (**d**) alloy D; (**e**) alloy E; (**f**) alloy F; (**g**) alloy G; (**h**) alloy H; (**i**) alloy I; (**j**) alloy J; (**k**) alloy K, and (**l**) alloy L.

**Figure 2:**
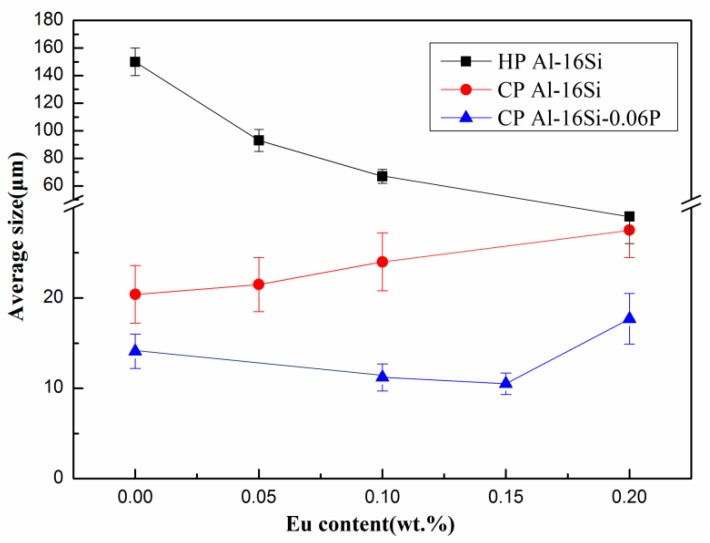
Average size of primary Si crystals in HP Al–16Si, CP Al–16Si, and CP Al–16Si–0.06P alloys with respect to the Eu addition levels.

Figure 1 depicts the microstructures of the three series of HP Al–16Si, CP Al–16Si, and CP Al–16Si–0.06P alloys with different Eu concentrations. Meanwhile, the relationship of the corresponding average sizes of primary Si and Eu concentration are illustrated in Figure 2. Coarse irregular primary Si with an average size of 149.3 µm and the plate–like eutectic Si were found in unmodified high purity (HP) Al–16Si alloys, as demonstrated in Figure 1a. Figure 1b–d and Figure 2 show that the primary Si crystals were refined gradually as the Eu content increased. In particular, when the Eu addition was 0.2%, the average size of primary Si lowered to 29.1 µm, showing a significant refining effect. At the same time, the eutectic Si was transformed into a fibrous structure (Figure 1d). The results show that the Eu element can simultaneously refine the primary Si and modify the eutectic Si in the HP Al–16Si alloy.

Figure 1e–h describe the SEM micrographs of the CP Al–16Si alloys with different Eu contents. Compared to the unmodified HP Al–16Si alloy, it was found that the primary Si crystals were greatly refined due to the presence of the P impurity element in the unmodified CP Al–16Si alloy, but the plate–like eutectic Si became thicker. Although the plate–to–fibrous transformation of eutectic Si was also found in CP Al–16Si alloys, the primary Si particles gradually became larger as the Eu content increased, as shown in Figure 2. In particular, when the Eu addition was 0.2%, the primary Si particles were much coarser (Figure 1h). The mutual poisoning between Eu and P in hypoeutectic Al–Si alloys was reported in our previous work [40], where the coarse Al_2_Si_2_Eu phase was believed to react with AlP. Moreover, the EuP phase was also expected to be preferentially formed [41]. Hence, the coarsening of primary Si in Eu–modified CP Al–16Si alloys appears to be caused by the depletion and poisoning of pre–existing AlP impurities in the melt. The results show that Eu cannot simultaneously refine the primary Si and modify the eutectic Si in the CP Al–16Si alloy.

Figure 1i–l demonstrate the microstructures of the CP Al–16Si–0.06P alloys containing various amounts of Eu. Compared to the unmodified CP Al–16Si alloy, the primary Si crystals had a smaller size in the unmodified CP Al–16Si–0.06P alloy, as shown in Figure 1i. Figure 1j,k show that the additions of 0.1% Eu and 0.15% Eu can further refine the primary Si on the basis of P refinement. When the Eu addition was 0.15%, the average size of primary Si was reduced to the minimum of 10.5 µm (Figure 1k), as shown in Figure 2. Nevertheless, by further increasing the Eu addition to 0.2%, the average size of the primary Si particles began to increase, and became even larger than the ones in the unmodified CP Al–16Si–0.06P alloys (Figure 1i), as shown in Figure 1l. With respect to eutectic Si, it was found that the shape was still coarse and plate–like in the unmodified CP Al–16Si–0.06P alloy (Figure 1i). The addition of 0.1% Eu produced the partial modification, where the plate–like eutectic Si and fibrous eutectic Si coexisted in the microstructure (Figure 1j). When we further increased the Eu additions to 0.15% and 0.2%, the fully modified fibrous eutectic Si was observed (Figure 1k,l). Therefore, the primary Si of the minimum average size with the fully fibrous eutectic Si was obtained in CP Al–16Si–0.06P alloys through the addition of 0.15% Eu.

In summary, simultaneous primary Si refinement and eutectic Si modification by Eu was obtained in the HP Al–16Si and CP Al–16Si–0.06P alloys, but not in the CP Al–16Si alloys, which indicates that the influence of Eu on the refinement efficiency of primary Si in hypereutectic Al–Si alloys is closely associated with the alloy purity.

### 3.2. Refinement Mechanism of Primary Si in the Eu–Modified HP Al–16Si Alloy

#### 3.2.1. 3D Morphologies of Primary Si

**Figure 3:**
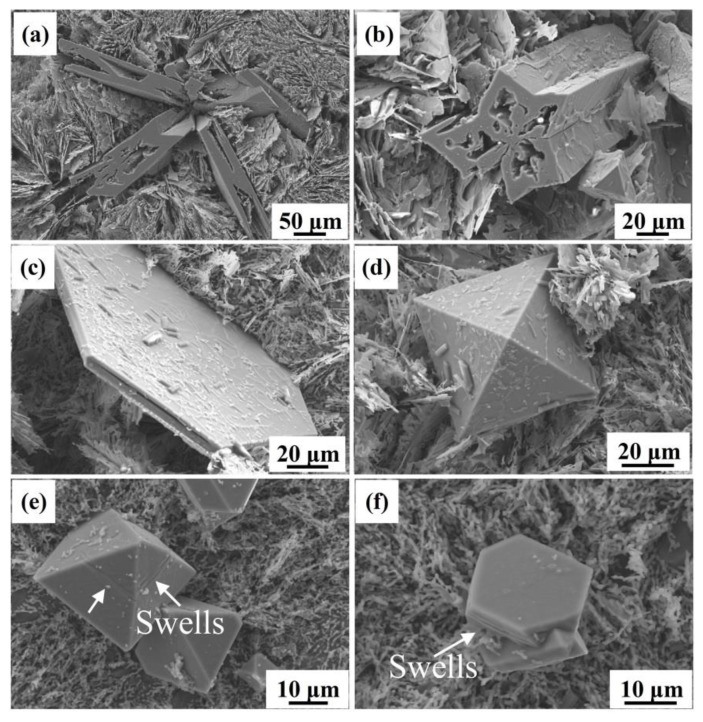
Effect of Eu contents on the 3D morphologies of primary Si in the HP Al–16Si alloys: (**a**–**d**) unmodified and (**e**,**f**) 0.2% Eu.

The typical 3D morphologies of primary Si in unmodified HP Al–16Si alloys are depicted in Figure 3. It was discovered that the unmodified coarse primary Si included five–branched (Figure 3a), five–star prismatic (Figure 3b), hexagonal plate–like (Figure 3c), and octahedral (Figure 3d) morphology. It was reported that the five–branched and five–star prismatic primary Si grew from twinned decahedron cores made up of five Si tetrahedrons [42]. The development in the five radial directions and the elongation of primary Si in the height direction can be well explained by the twin plane re–entrant edge (TPRE) mechanism [42,43], which postulates that a stable groove of 141° exists between the twinned planes and Si has a quicker growth rate at the groove along the <112> growth direction of Si. Furthermore, the hexagonal plate–like primary Si was believed to grow from two Si tetrahedrons in a twin relationship, whose growth can be also interpreted by the TPRE mechanism [44].

When the Eu addition was 0.2%, the coarse five–branched primary Si in the unmodified alloy vanished entirely and the number of five–star prismatic primary Si was also greatly reduced. Most of the primary Si particles were octahedral (Figure 3e) and had a hexagonal plate–like (Figure 3f) morphology with decreased average size, indicating that the TPRE mechanism is also valid for primary Si in Eu–modified HP Al–16Si alloys. It is a remarkable fact that the swells were regularly found at the surface of primary Si (Figure 3e,f), which could be due to the transformation from planar growth to cellular growth of primary Si under the undercooling condition [45].

#### 3.2.2. Thermal Analysis

**Figure 4:**
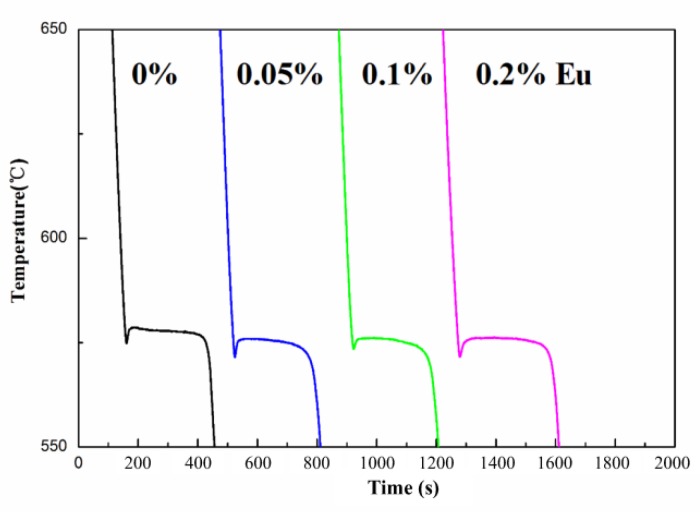
Effect of Eu content on the cooling curves of the HP Al–16Si alloy.

Figure 4 depicts the cooling curves of HP Al–16Si alloys with varying Eu content. Only the Al–Si eutectic reaction can be distinguished in the cooling curves of the unmodified HP Al–16Si alloys. The insufficient heterogeneous nucleating substrates in HP Al–16Si alloys restrict the precipitation of primary Si, even at a temperature near the Al–Si eutectic reaction, leading to the disappearance of slope changes for the formation of primary Si in cooling curves. However, the introduced Eu element had no influence on the formation of primary Si, showing that the refinement mechanism of primary Si by Eu is not a heterogeneous nucleation mechanism like that of AlP.

#### 3.2.3. Eu Distribution in HP Al–16Si Alloy

**Figure 5:**
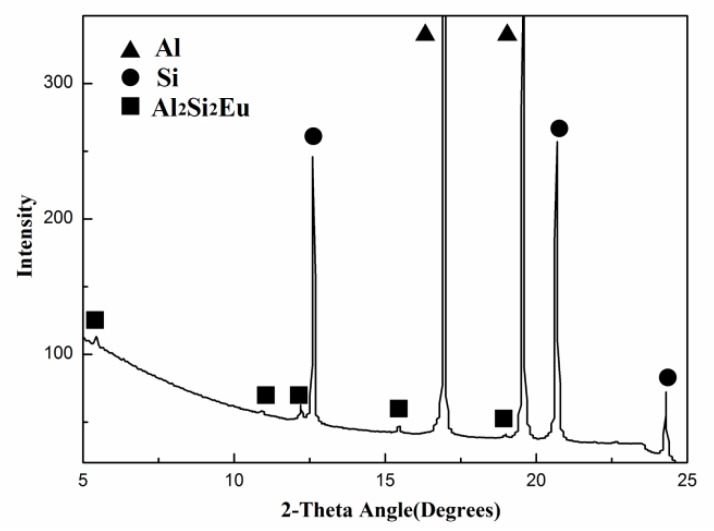
Micro x–ray diffraction pattern of the 0.2% Eu–modified HP Al–16Si alloy.

**Figure 6:**
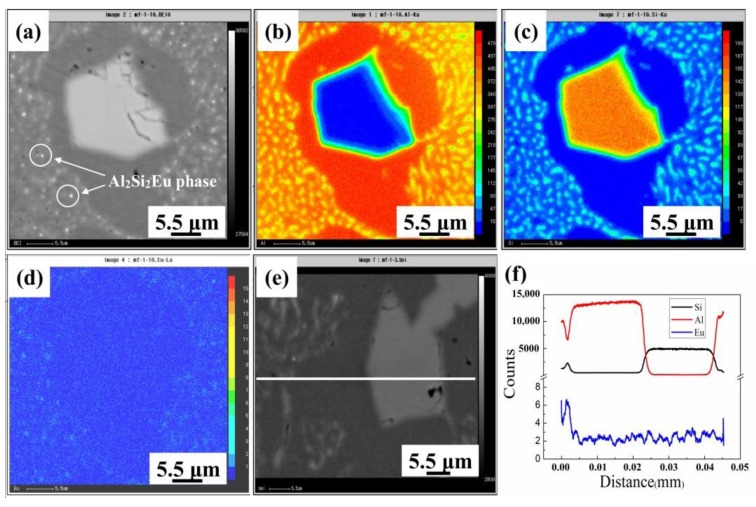
Electron probe microanalysis (EPMA) mappings of primary Si in the 0.2% Eu–modified HP Al–16Si alloy: (**a**) map of the back scattered electron (BSE) image; (**b**) Al mapping; (**c**) Si mapping; (**d**) Eu mapping; (**e**) line analysis of the BSE image; (**f**) line analysis of the Al, Si, and Eu elements.

For purpose of elucidating the Eu distribution in the microstructure, Figure 5 presents the micro x–ray diffraction (μ–XRD) pattern for the HP Al–16Si–0.2Eu alloy, confirming the existence of the Al_2_Si_2_Eu phase together with Al and Si. The overall distribution of Eu in the HP Al–16Si–0.2Eu alloy was easy to distinguish in the back scattered electron (BSE) image with a much brighter contrast in the microstructure, where the small Al_2_Si_2_Eu phase was frequently observed in the eutectic mixture, as demonstrated in Figure 6a. The electron probe microanalysis (EPMA) mappings in Figure 6b–d show that no distinct enrichment of Eu was observed within primary Si, and Eu was mainly distributed in the eutectic mixture, which corresponded well to the small Al_2_Si_2_Eu phase in Figure 6a. It was further supported by using the EPMA line analysis in Figure 6e,f, indicating that the Eu concentration in eutectic Si was much higher than primary Si.

#### 3.2.4. Transmission Electron Microscopy Observation

As is widely known, the growths of plate–like eutectic Si and most of the unmodified primary Si phase are facilitated by the TPRE mechanism. Therefore, few parallel Si twins grown on one special plane, rather than multiple twins, were frequently observed in unmodified Si phase [19,20]. The well–established growth mode of Si after modification includes the poisoning of the TPRE mechanism [44] and impurity induced twinning (IIT) mechanism [46]. The poisoning of the TPRE mechanism presumes that the modifier atoms restrict Si phase growth by selectively adsorbing at TPRE, thereby removing the growth advantages of Si at TPRE. The IIT mechanism assumes that the modifier atoms can be adsorbed on the growing {111}_Si_ planes, thus generating frequent multiple Si twins. It should be emphasized that either the poisoning of the TPRE mechanism or ITT mechanism can be related to the absorption of modifier atoms in the Si phase.

**Figure 7:**
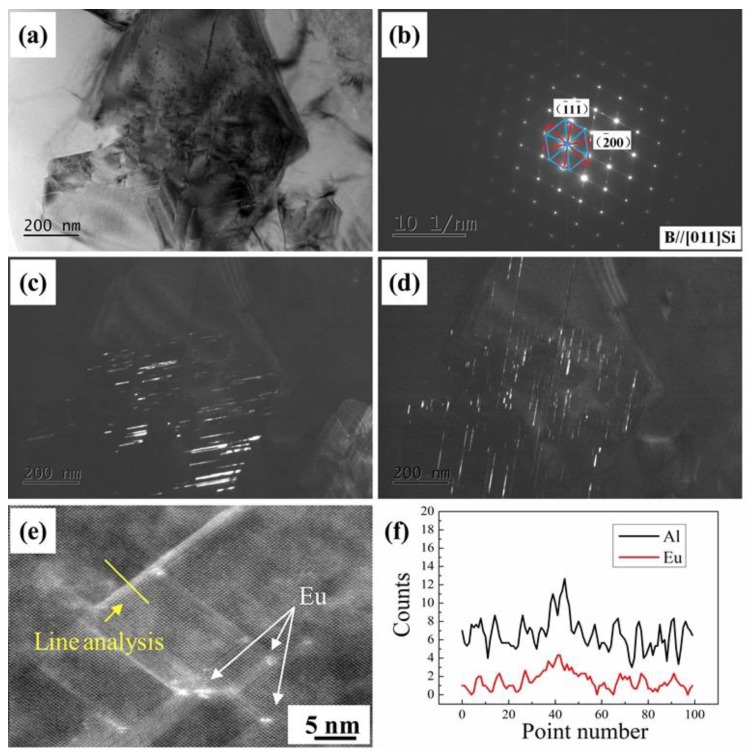
Transmission electron microscopy (TEM) image of eutectic Si with intersecting twins in the 0.2% Eu–modified HP Al–16Si alloy: (**a**) TEM BF image; (**b**) corresponding selected area diffraction pattern; (**c**,**d**) central DF images taken from the two {111}_Si_ spots of the two variants in (**b**); (**e**) high–angle annular dark–field scanning transmission electron microscopy (HAADF–STEM) image of eutectic Si and (**f**) line analysis of the Al and Eu elements in (**e**).

Figure 7 displays a eutectic Si particle in the HP Al–16Si–0.2Eu alloy, which was tilted to the principal twinning orientation of Si (<110>) to observe the Si twin. It can be clearly observed in Figure 7a that most eutectic Si particles were multiple twinned. The corresponding selected area diffraction pattern (SADP) with the double diffraction of two variants in Figure 7b indicates that the Si twin grew along the {111}_Si_ plane. The centered dark–field images derived from two diffraction spots corresponded to two distinct variants, which grew along the <112>_Si_ directions with an angle of 70.5° between them, as shown in Figure 7c,d. The high–angle annular dark–field scanning transmission electron microscopy (HAADF–STEM) image and its line analysis show that the Eu element was discovered along the <112> growth direction of eutectic Si and at the intersection of two {111}_Si_ twins, demonstrating that the IIT mechanism and poisoning of the TPRE mechanism of Eu are valid.

**Figure 8:**
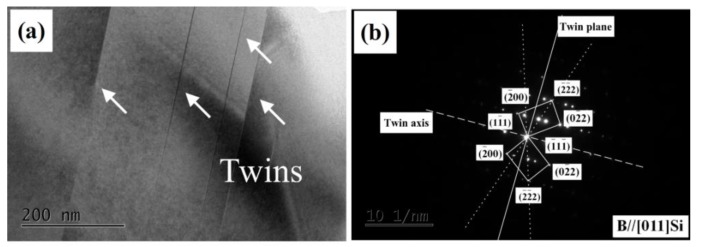
TEM image of primary Si in the 0.2% Eu–modified HP Al–16Si alloy: (**a**) TEM bright–field image; (**b**) corresponding selected area diffraction pattern.

However, unlike eutectic Si, the Eu element did not greatly partition into the primary Si and surrounding Al dendrites in Figure 6d. Compared to the modified eutectic Si (Figure 7), fewer parallel twins, rather than multiple twins, were observed in primary Si in the HP Al–16Si–0.2Eu alloy, as shown in the TEM image of primary Si (Figure 8a) and the corresponding SADP (Figure 8b). Therefore, the refinement of primary Si in the HP Al–16Si–0.2Eu alloy is not caused by IIT and the poisoning of TPRE mechanisms. As Eu has exceedingly limited solid solubility in primary Si, the accumulation of Eu elements at the growing interface of primary Si will be formed due to the rejection of the Eu solute from the primary Si during the solidification process. Therefore, a constitutional undercooling was established, which restricted the growth of primary Si and refined them in the HP Al–16Si–0.2Eu alloy.

In conclusion, the refinement of primary Si in the HP Al–16Si alloy is caused by the constitutional undercooling of Eu. The different modification mechanisms of Eu on primary Si and eutectic Si could be due to their different growth conditions, resulting in different interfacial adsorption performance of the Eu element in the Si phase.

### 3.3. Refinement Mechanism of Primary Si in the Eu–Modified CP Al–16Si–0.06P Alloy

#### 3.3.1. Thermal Analysis

**Figure 9:**
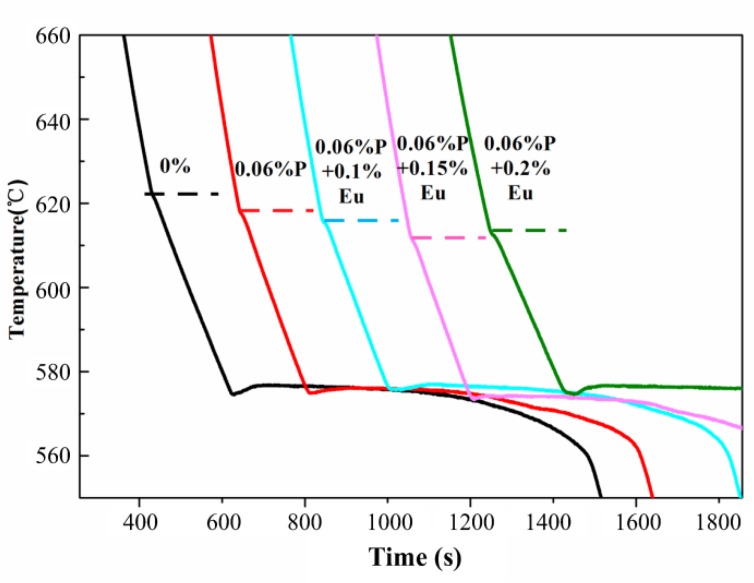
Effect of Eu contents on the cooling curves of the CP Al–16Si–0.06P alloy.

Figure 9 presents the measured cooling curves of unmodified and Eu–modified CP Al–16Si–0.06P alloys. Due to the existence of AlP impurities in CP Al–16Si alloys, the formation of primary Si appears in the cooling curves and the corresponding primary Si reaction temperatures are shown in Table 2. The primary Si reaction temperature was discovered to be reduced by the addition of 0.06% P, which agreed well with the research by Liu Y. et al. [47], who pointed out that the cooling curves hardly indicated the nucleation of primary Si. The small volume fraction of primary Si results in the little released latent heat, which cannot alter the slope until very late after the nucleation of primary Si. Thus for primary Si, the slope variation responds more to growth than nucleation, and the depressed slope variation in the Al–16Si–0.06P alloy actually indicates the slow growth of primary Si restricted by AlP [47]. With the addition of 0.1% Eu and 0.15% Eu to the CP Al–16Si–0.06P alloys, the slope changes for primary Si were further reduced, indicating that the growth of primary Si is hindered by Eu. However, the primary Si reaction temperature began to increase with the addition of 0.2% Eu, which suggests that the refinement mechanism of primary Si changed in this situation.

#### 3.3.2. Primary Si Nucleus in the CP Al–16Si–0.06P Alloy

**Figure 10:**
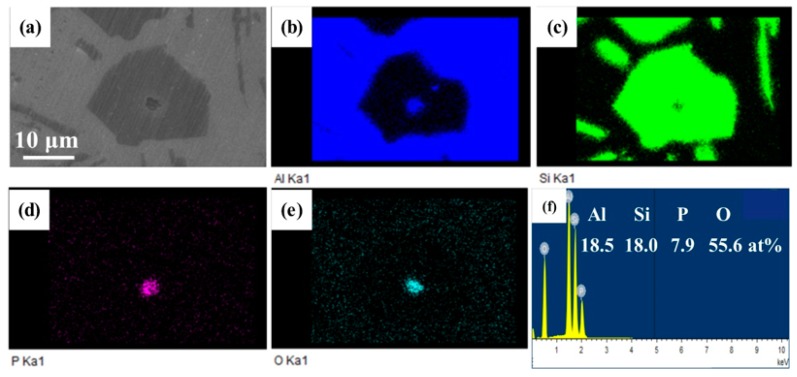
Energy dispersive x–ray spectroscopy (EDX) analysis of primary Si particles in the 0.15% Eu–modified CP Al–16Si–0.06P alloy: (**a**) SEM image; (**b**) Al; (**c**) Si; (**d**) P; (**e**) O, and (**f**) EDX point analysis of black particle in (**a**).

Figure 10 shows the energy dispersive x–ray spectroscopy (EDX) mapping of a primary Si in the 0.15% Eu–modified Al–16Si–0.06P alloy. It can be clearly observed that the nucleus of primary Si contained Al, P, and O elements. K. Nogita et al. [13] reported the orientation relationships between the nucleus and Si by TEM and identified it as AlP. It is worth mentioning that no intermetallic formation between Eu and P was observed in the microstructure, which proves that all of the AlP particles can still act as a heterogeneous nucleating substrate for primary Si in the CP Al–16Si–0.06P alloy with the addition of 0.15% Eu.

**Figure 11:**
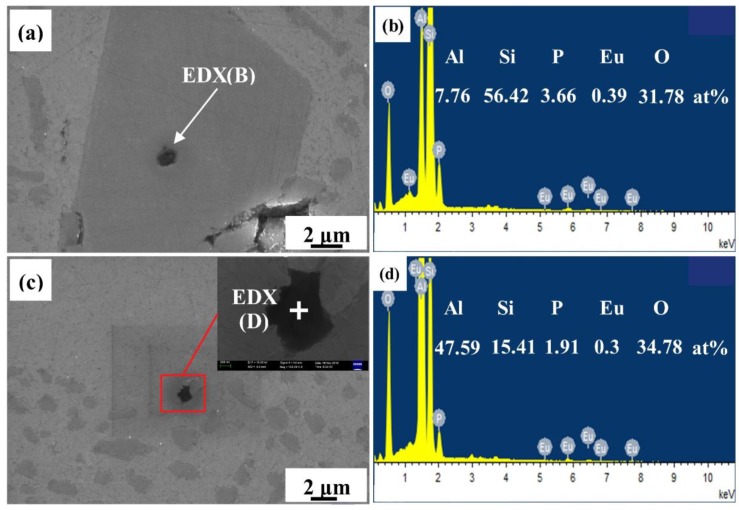
SEM images of the P enriched black particles in the 0.2% Eu–modified CP Al–16Si–0.06P alloy: (**a**) P enriched black particle within primary Si; (**b**) EDX point analysis of black particle in (**a**); (**c**) P enriched black particle within Al–Si eutectic; and (**d**) EDX point analysis of black particle in (**c**).

Figure 11 depicts the EDX analysis of a primary Si in the 0.2% Eu–modified Al–16Si–0.06P alloy. Aside from the prospective intensity signals for Al, P, and O, the presence of Eu was found in the nucleus of primary Si, as shown in Figure 11b. Meanwhile, few similar black particles containing Al, P, O, and Eu were also discovered in the eutectic mixture, as depicted in Figure 11c,d. However, there was no primary Si nucleating at these particles. This indicates that Eu may solubilize in AlP to form (Al, Eu)P compounds and weaken the ability of AlP as the heterogeneous nucleating substrate for primary Si, which is consistent with the results of the microstructures (Figure 1) and cooling curves (Figure 9).

#### 3.3.3. Solidification of the CP Al–16Si–0.06P Alloy

**Figure 12:**
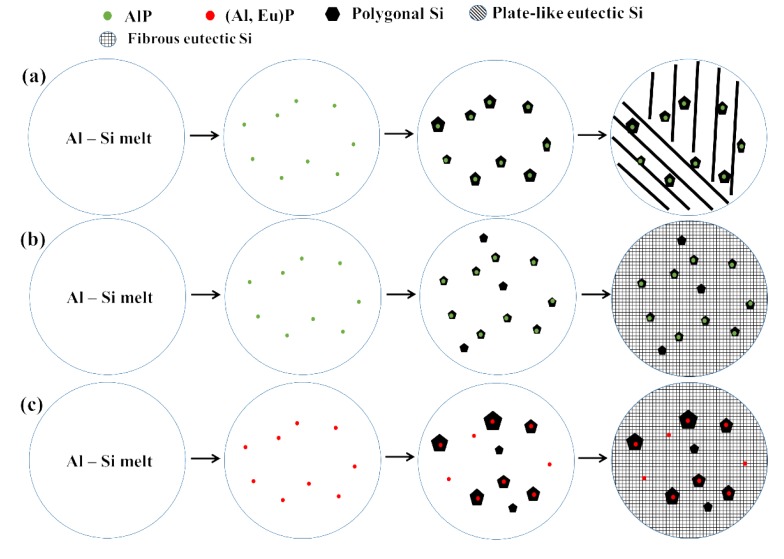
Effect of Eu content on the solidification processes and refinement mechanisms of CP Al–16Si–0.06P alloys: (**a**) unmodified; (**b**) 0.1% and 0.15% Eu; (**c**) 0.2% Eu.

The possible solidification processes and refinement mechanisms of primary Si in CP Al–16Si–0.06P alloys with varying Eu content can be schematically illustrated in Figure 12.

(1) After 0.06% P is added, the AlP phase will precipitate at a higher temperature. With a drop in temperature during solidification, the AlP crystals can nucleate primary Si. The refinement of primary Si in the unmodified Al–16Si–0.06P alloy can be achieved given the heterogeneous nucleation mechanism of AlP, as depicted in Figure 12a. However, the eutectic Si is still plate–like.

(2) When adding 0.1% Eu and 0.15% Eu to the CP Al–16Si–0.06P alloys, all of the AlP crystals can still act as the heterogeneous nucleating substrate for primary Si. Moreover, the primary Si is further refined by Eu due to the formation of an Eu solute enrichment layer near the liquid–solid interfaces of primary Si, which creates higher constitutional supercooling and hinders the growth of primary Si. As the temperature reduces to the eutectic temperature, the eutectic Si is modified to a fibrous morphology by Eu simultaneously. Therefore, the refinement of primary Si is caused by the heterogeneous nucleation mechanism of AlP combined with the constitutional supercooling mechanism of Eu.

(3) By further increasing Eu additions to 0.2%, part of the AlP particles are poisoned due to the formation of (Al, Eu)P. Although the constitutional supercooling caused by Eu still exists, the influence of the poisoning of AlP crystals on the size of primary Si is more remarkable, leading to an increase in the average size of primary Si. Thus at this point, the average size of primary Si is affected by the heterogeneous nucleation mechanism of AlP, the constitutional supercooling mechanism of Eu, and the poisoning of AlP.

In summary, to refine the primary Si in the CP hypereutectic Al–16Si–0.06P alloy, the Eu:P weight ratio should not exceed 3.33. Otherwise, the refinement efficiency of primary Si will be reduced due to the mutual poisoning between Eu and P. This can also be used to explain the coarsening of primary Si in Eu–modified CP Al–16Si alloys containing 26 ppm P impurity, where the Eu:P weight ratio is easily exceeds 3.33 by simply adding a small amount of Eu.

### 3.4. Mechanical Properties of the Eu–Modified CP Al–16Si–0.06P Alloy

**Figure 13:**
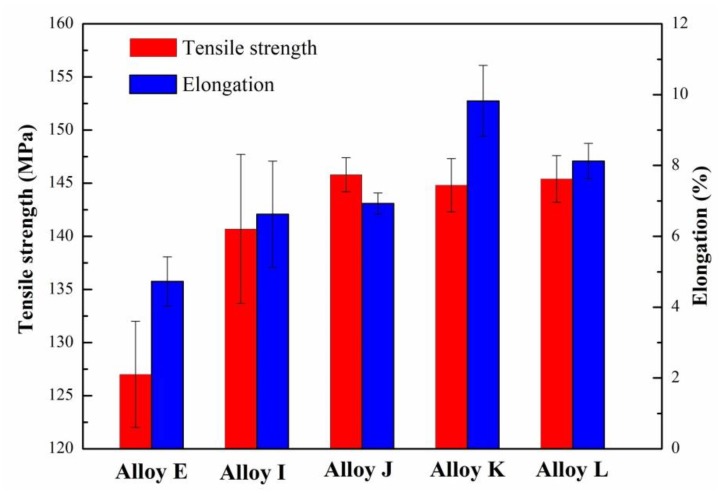
Tensile properties of the CP hypereutectic Al–16Si–0.06P alloys with different Eu content.

Figure 13 presents the mechanical properties including the ultimate tensile strength (UTS) and elongation (EI) of the CP Al–16Si–0.06P alloys with varying Eu contents. It is evident that the UTS and EI of the CP Al–16Si alloy were enhanced after the addition of 0.06% P. The addition of some extra Eu elements further improved both the UTS and EI. Compared to the CP Al–16Si–0.06P alloy, the UTS was enhanced by 3% from 140.7 MPa to 144.8 MPa, while the EI was increased by 48% from 6.6% to 9.8% through the addition of 0.15% Eu. However, further increasing the Eu addition to 0.2% led to a decrease in both the UTS and EI of the CP Al–16Si–0.06P alloys.

**Figure 14:**
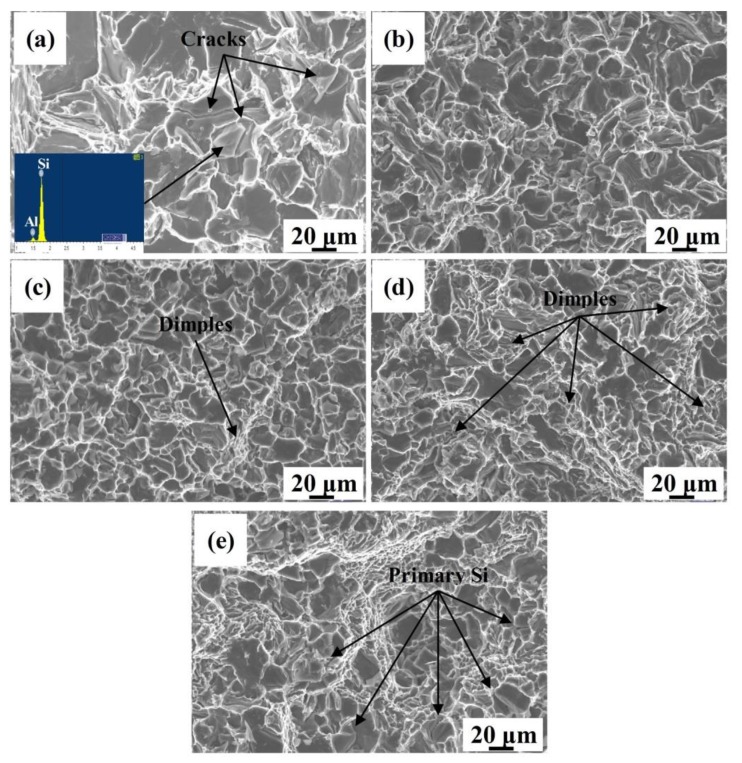
The fracture surfaces of the CP hypereutectic Al–16Si–0.06P alloys with various additions of Eu: (**a**) Al–16Si; (**b**) Al–16Si–0.06P; (**c**) Al–16Si–0.06P–0.1Eu; (**d**) Al–16Si–0.06P–0.15Eu, and (**e**) Al–16Si–0.06P–0.2Eu.

Figure 14 presents the fracture surfaces of CP hypereutectic Al–16Si–0.06P alloys with various additions of Eu. The fracture surface of the CP Al–16Si alloy was found to be mainly covered by the cleavage plane, demonstrating a clear brittle fracture nature due to coarse primary Si and plate–like eutectic Si in the matrix, as presented in Figure 14a. In addition, cracked primary Si particles—proven through EDX analysis in Figure 14a—were also frequently observed on the fracture surface. Figure 14b shows that the fracture surface of the CP Al–16Si–0.06P alloy. It was discovered that the number of cracked primary Si and the cleavage planes were reduced due to primary Si refinement. When adding 0.1% Eu to Al–16Si–0.06P alloys, some dimples formed on the fracture surfaces in Figure 11c, which can be due to the further refinement of primary Si and partial modification of eutectic Si. With the addition of 0.15% Eu, the smallest primary Si and fully modified eutectic Si were obtained. Hence, more and smaller dimples were found on the fracture surface, corresponding to excellent plasticity, which shows that the fracture was diverted from a brittle fracture to a mixed ductile–brittle fracture. Further increasing the Eu content to 0.2%, some cracked primary Si particles were again found on the fracture surface due to the increase in size of primary Si, as illustrated in Figure 14e. According to the report, the fracture mechanism of Al–Si alloys is primarily related to three aspects: (a) the size and distribution of the Si phase; (b) the cohesion between the Si phase and matrix; and (c) the fracture of the Si phase [3]. The relationship between the intrinsic fracture stress (*σ_f_*) on the Si particles and the internal defect length (*C*) is provided by the Griffith equation [48,49]:

(2) $$\sigma_{f}={\left(\frac{2E\gamma }{\pi C}\right)}^{1/2}$$

where *γ* is the fracture surface energy and *E* is the Young’s modules of particle. On the basis of the Griffith equation, coarse Si crystals have a lower intrinsic fracture stress (*σ_f_*) because the internal defects of coarse Si crystals are longer than fine Si crystals. As a result, the coarse primary Si and eutectic Si in the unmodified CP Al–16Si alloy will be easier to fracture under the tensile test. Furthermore, plate–like eutectic Si possess incisive edges and corners, which are stress concentration and crack initiation sites. However, the crack tips will be inactivated by fibrous eutectic Si, which restrains a further cleavage [50]. Hence, Eu modification can evidently enhance the UTS and EI of CP hypereutectic Al–16Si–0.06P alloys.

## 4. Conclusions

Different influences of the addition of Eu on primary Si refinement in varied purity hypereutectic Al–Si alloys were researched and the following conclusions obtained:

(1) The P impurity element in hypereutectic Al–Si alloys has great influence on the rare earths’ refinement efficiency of primary Si. The simultaneous primary Si refinement and eutectic Si modification by Eu was obtained in the HP Al–16Si and CP Al–16Si–0.06P alloys, but the primary Si was gradually coarsened in the CP Al–16Si alloys.

(2) The refinement of primary Si in the HP Al–16Si alloys was caused by the constitutional undercooling of Eu. There was no sufficient Eu element partition into the primary Si particles and fewer parallel twins, rather than multiple twins, were observed within them.

(3) The refinement of primary Si in the CP Al–16Si–0.06P alloys was caused by the overlay of two kinds of mechanisms including the heterogeneous nucleation mechanism of AlP and the constitutional supercooling mechanism of Eu. However, in order to refine the primary Si in the CP hypereutectic Al–16Si alloys, the Eu:P weight ratio should not exceed 3.33, otherwise the refinement efficiency of primary Si will be reduced due to mutual poisoning between Eu and P 

(4) An excellent integration of ultimate tensile strength (144.8 MPa) and elongation (9.8%) of the CP hypereutectic Al–16Si–0.06P alloy was obtained by adding 0.15% Eu.

## Figures and Tables

**Table 1:** Alloy compositions of hypereutectic Al–16Si alloys in the present experiments. The given values of the Eu addition are nominal.

Sample	Purity	Si (wt. %)	Fe (wt. %)	P (ppm)	Al	Eu Addition (wt. %)
Alloy A	HP	16	<0.01	0.5	Balance	0
Alloy B						0.05
Alloy C						0.1
Alloy D						0.2
Alloy E	CP	16	0.13	26	Balance	0
Alloy F						0.05
Alloy G						0.1
Alloy H						0.2
Alloy I	CP	16	0.13	594	Balance	0
Alloy J						0.1
Alloy K						0.15
Alloy L						0.2

**Table 2:** Effect of Eu contents on the primary Si reaction temperatures in the cooling curves of the CP Al–16Si–0.06P alloys.

Alloys	Alloy E	Alloy I	Alloy J	Alloy K	Alloy L
Primary Si reaction temperature (°C)	621.7	617.9	615.2	611.5	612.5
